# Supplementary material for: Characterization of Odor-Active Compounds from Gryllus bimaculatus Using Gas Chromatography-Mass Spectrometry-Olfactometry
Source: Foods. 2023 Jun 9;12(12):2328. doi: 10.3390/foods12122328 (PMC10297462; doi:10.3390/foods12122328)
Supplement: Supplementary file 1 [file foods-12-02328-s001.zip › foods-2430653-supplementary.pdf]

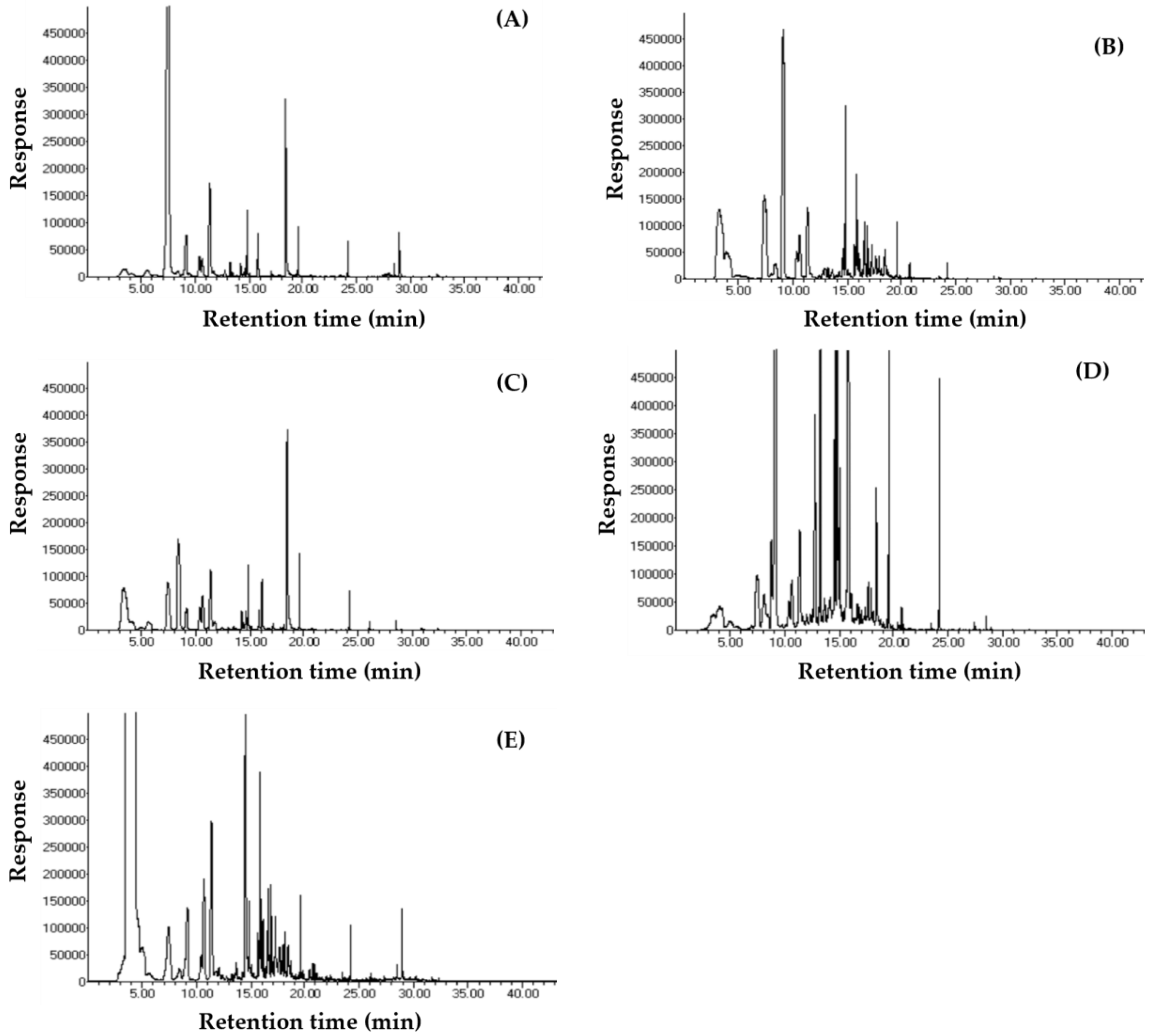

Figure S1. Total ion chromatograms. (A) control, (B) hot drying, (C) freeze drying, (D) auto clave and (E) de-fatted, respectively.

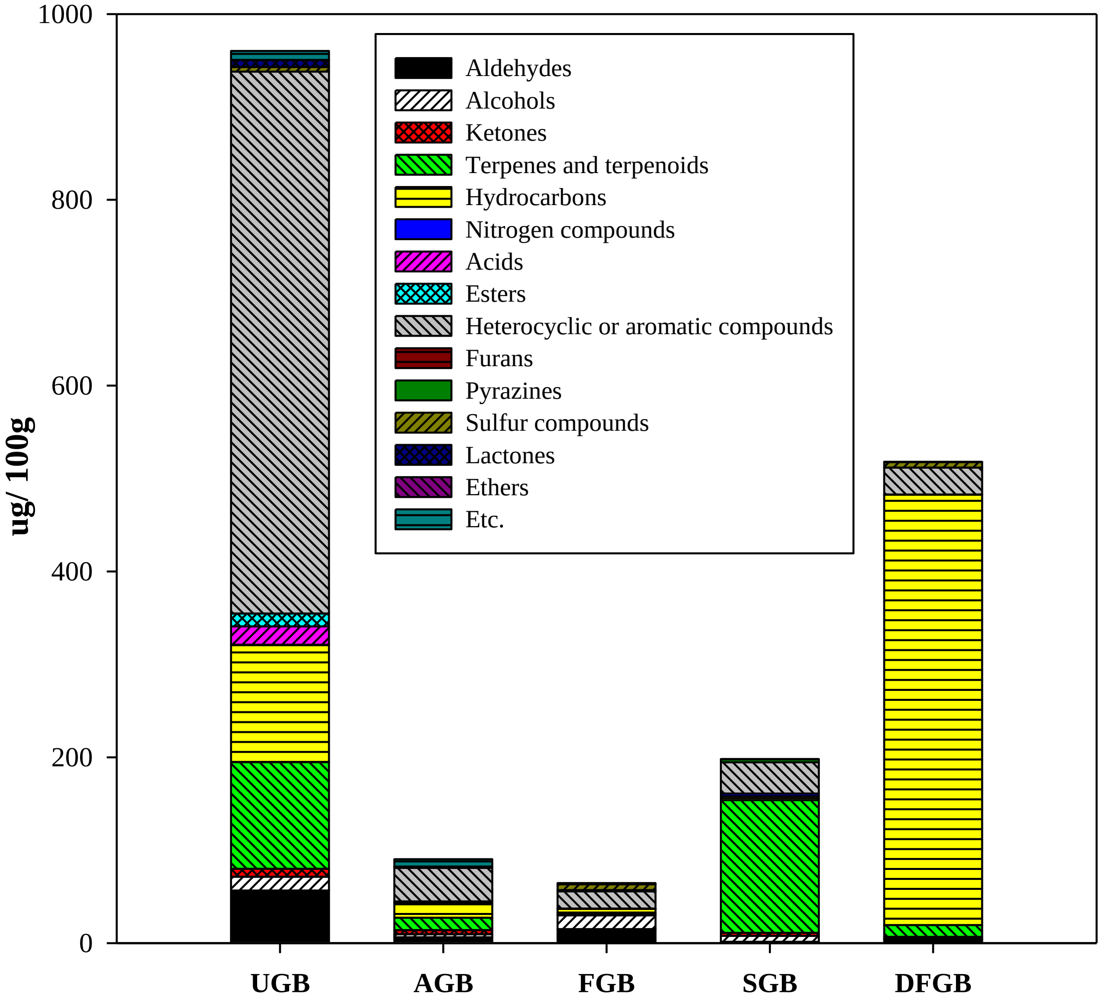

Figure S2. Composition of volatile compounds in five kinds of GB.
